# A network meta-analysis of acupuncture therapy for female insomnia and negative emotions from the perspective of the perimenopausal window

**DOI:** 10.3389/fneur.2025.1726927

**Published:** 2026-01-23

**Authors:** Shumin Wang, Linlin Bai, Pengyan Zhu, Hailong Wang, E. Zhou, Miaomiao Jing, Shuai Fu, Qin Lyu, Tianyu Bai

**Affiliations:** 1 Department of Acupuncture and Massage, Shandong Provincial Third Hospital, Shandong University, Jinan, Shangdong, China; 2 Graduate School of Shandong Sports College, Jinan, Shandong, China; 3 Liaocheng Hospital of Traditional Chinese Medicine, Liaocheng, Shandong, China; 4 Department of Acupuncture and Moxibustion, Yantai Hospital of Traditional Chinese Medicine, Yantai, Shandong, China; 5 Jining Hospital of Xiyuan Hospital of China Academy of Chinese Medical Sciences, Jining, Shandong, China

**Keywords:** acupuncture, insomnia, negative emotions, network meta-analysis, perimenopause

## Abstract

**Background:**

Perimenopausal women frequently experience insomnia and negative emotions due to hormonal fluctuations. Acupuncture, a traditional Chinese therapy, has attracted significant interest for its potential to regulate endocrine function and alleviate insomnia. Despite this, no systematic review has hitherto evaluated the efficacy of acupuncture on insomnia and negative emotions in perimenopausal women. This network meta-analysis was conducted to assess the therapeutic effects of acupuncture on these conditions, thereby generating robust clinical evidence to inform evidence-based practice and guide future research directions.

**Methods:**

A systematic literature search was performed in multiple databases, such as PubMed, Web of Science, Medline, Scopus, Wanfang, CNKI, VIP Database, and CBM, covering all records from inception through November 2025. The primary outcome was measured using the Pittsburgh Sleep Quality Index (PSQI), while secondary outcomes were evaluated through various depression and anxiety scales, including the Kupperman Menopausal Index, Hamilton Anxiety Rating Scale, Hamilton Depression Rating Scale, Self-Rating Anxiety Scale, Self-Rating Depression Scale, Generalized Anxiety Disorder-7, Patient Health Questionnaire-9, Beck Depression Inventory, and Beck Anxiety Inventory.

**Results:**

According to the network meta-analysis, the top three interventions identified for the improvement of PSQI scores were, in order: routine acupuncture combined with auricular acupuncture; auricular acupuncture combined with Western medicine; and routine acupuncture combined with Pentatonic therapy. Seven interventions demonstrated significant effects compared to SH (*P* < 0.05). Regarding negative moods, balance acupuncture combined with Xiaoyao powder, routine acupuncture combined with pentatonic therapy, and abdominal acupuncture combined with sedative prescription and western medicine ranked highest (*P* < 0.05).

**Conclusion:**

This network meta-analysis suggests that routine acupuncture combined with auricular acupuncture may be an effective intervention treatment for treating insomnia in perimenopausal women. Furthermore, balanced acupuncture combined with Xiaoyao powder may have a positive effect in alleviating negative emotions.

## Introduction

Menopause, also known as perimenopause, represents a natural transition denoting the cessation of ovarian function and has been recognized as a critical period of heightened vulnerability for the co-occurrence of insomnia and depression (1–3). Epidemiological data suggest that approximately 50% of women aged 40 to 64 worldwide experience sleep disturbances and mood disorders during this phase (4, 5). Anxiety and depressive symptoms play a key mediating role in the relationship between hot flashes, sweating, and the decline in sleep quality. Current evidence suggests that anxiety symptoms account for 17.86% of this indirect effect, while depressive symptoms contribute an additional 5.36% (6). Furthermore, women with pre-existing insomnia are at significantly higher risk for moderate to severe insomnia during the perimenopausal and postmenopausal stages (7). However, current treatment options remain limited. Benzodiazepines and non-benzodiazepine medications, although effective in rapidly alleviating insomnia symptoms by modulating the gamma-aminobutyric acid (GABA) system, may lead to adverse effects such as daytime drowsiness, cognitive impairments, motor dysfunction, and drug dependence with long-term use (8, 9). Therefore, there is an urgent need to develop comprehensive treatment strategies that combine neurobiological regulation with psychological interventions.

In recent years, a growing body of molecular biology evidence has substantiated the significant potential of acupuncture in the treatment of psychiatric and sleep-related disorders. Interestingly, studies have shown that acupuncture, through the stimulation of acupoints, can modulate various neurotransmitters such as melatonin, norepinephrine, endogenous opioids, and GABA, thereby improving sleep quality and alleviating depressive symptoms (10–12). The fundamental principles of traditional Chinese medicine involve the stimulation of specific acupoints to regulate the balance of Yin and Yang, helping the body return to an optimal physiological state (13). For instance, Baihui (GV20) has been shown to effectively enhance brain connectivity and reduce abnormal neural activity, improving sleep quality (14). Furthermore, combining electrical stimulation with traditional acupuncture yields more significant therapeutic effects (15). However, systematic evidence from evidence-based medicine remains scarce. Therefore, this study sought to explore the application and efficacy of clinical acupuncture in the treatment of peri-menopausal insomnia and its comorbid depression, addressing the urgent need for comprehensive treatment strategies that combine neurobiological regulation and psychological interventions to guide symptom relief and long-term prognosis optimization.

## Methods

### Study registration

The study protocol underwent prospective registration with PROSPERO (CRD42025630058), where full documentation is publicly accessible (16). As no primary data were collected, formal ethical approval was not required.

### Strategy for literature search

We systematically searched PubMed, Web of Science, Scopus, MEDLINE, Wanfang, China National Knowledge Infrastructure (CNKI), VIP Database, and Chinese Biomedical Literature Database (CBM) using a predefined strategy. The primary search terms utilized were “Insomnia”, “Perimenopause”, and “Acupuncture Therapy”. The specific search strategies are delineated in Table 1.

**Table 1:** PubMed search methodology.

**No**	**Search items**
#1	Acupuncture therapy. Mesh
#2	Navel acupuncture. ti. ab
#3	Pharmacoacupuncture treatment. ti. ab
#4	Acupotomy. ti. ab
#5	Electroacupuncture ti. ab
#6	Body acupuncture ti. ab
#7	Manual acupuncture ti. ab
#8	Electro-acupuncture ti. ab
#9	Auricular acupuncture ti. ab
#10	Laser acupuncture ti. ab
#11	Warm needling ti. ab
#12	Scalp acupuncture ti. ab
#13	Navel acupuncture ti. ab
#14	1 or 2–13
#15	Randomized controlled trial. Mesh
#16	Controlled clinical trial. ti. ab
#17	Randomized. ti. ab
#18	Randomly. ti. ab
#19	Trial. ti. ab
#20	15 or 16–19
#21	Sleep initiation and maintenance disorders. Mesh
#22	Early awakening. ti. ab
#23	Primary insomnia. ti. ab
#24	Insomnia. ti. ab
#25	Rebound insomnia. ti. ab
#26	21 or 22–25
#27	Menopause. Mesh
#28	Climacteric. ti. ab
#29	Perimenopause. ti. ab
#30	27 or 28–39
#31	#14 and #20 and #26 and #30

### Inclusion criteria

Literature screening was performed independently by two reviewers according to predetermined criteria. The screening and subsequent data extraction were guided by the PICOS framework (Population, Intervention, Comparison, Outcomes, Study design), ensuring a rigorous and structured approach. Only randomized controlled trials RCTs fulfilling the predefined eligibility criteria were incorporated into the final analysis. The inclusion criteria for this study are outlined using the P-I-C-O-S structure as summarized below.

#### Population

Participants who met all of the following criteria were eligible for inclusion.

A: Individuals within the age range of 45–55 years.

B: Perimenopausal stage defined by the STRAW criteria (17).

C: Insomnia disorder diagnosed according to the International Classification of Sleep Disorders, 3rd edition (ICSD-3) (18).

D: Irregular menstruation occurring in conjunction with symptoms including hot flashes, sweating, and mood disturbances.

E: No restrictions were applied regarding sex, race, socioeconomic status, ethnicity, or insomnia disorder severity.

#### Intervention

Eligible experimental interventions comprised various acupuncture modalities, including Routine acupuncture (RA), Auricular acupuncture (AA), Abdominal acupuncture (ABA), Electric acupuncture (EA), Scalp acupuncture (SA), Tongue acupuncture (TA), Ear point embedding seeds (EM), and Acupoint catgut embedding (ACE).

#### Comparator

Patients in the control group received one of several comparator interventions, including sham acupuncture, pharmacological treatment (traditional Chinese medicine and Western medicine), health education and lifestyle intervention (HI), pentatonic therapy (PT), and others. Any intervention that did not stimulate acupuncture points was excluded.

#### Outcomes

A: The primary outcome measure used for assessing sleep quality was the Pittsburgh Sleep Quality Index (PSQI).

B: The secondary outcome measures used to evaluate treatment efficacy comprised scales for depression and anxiety, including the Kupperman Menopausal Index (KMI), Hamilton Anxiety Rating Scale (HAMA), Hamilton Depression Rating Scale (HAMD), Self-Rating Anxiety Scale (SAS), Self-Rating Depression Scale (SDS), Generalized Anxiety Disorder 7-item Scale (GAD-7), Patient Health Questionnaire-9 (PHQ-9), Beck Depression Inventory (BDI), and Beck Anxiety Inventory (BAI).

#### Study design

Eligibility was restricted to RCTs to ensure the methodological rigor provided by random allocation of participants to experimental or comparator groups.

### Exclusion criteria

A: Patients who did not meet the menopause or age criteria were excluded.

B: Participants with an allergic diathesis to medications or a diagnosis of major systemic disorders were excluded.

C: Studies involving animals, quasi-RCTs, case reports, expert opinions, conference abstracts, and duplicate publications were excluded from the analysis.

D: Studies with unavailable or invalid data were excluded.

E: Trials that have missing primary outcome measures were excluded.

### Study selection and data extraction

A systematic screening process was conducted following the removal of duplicates. The process consisted of an initial title/abstract screening phase to exclude irrelevant records, followed by a full-text appraisal of remaining articles. Any uncertainties regarding eligibility were resolved by directly contacting the study authors.

Data extraction encompassed various parameters, including the first author's name, publication year, sample size, key study characteristics, intervention and control measures, intervention duration, outcome measures, and relevant data points. Throughout this process, both researchers independently executed the literature screening and data extraction, ensuring rigor through cross-verification against predefined inclusion and exclusion criteria. Any discrepancies that arose were referred to a third reviewer for arbitration, and consensus was achieved through thorough discussion.

### Evaluation of bias risk

The risk of bias for included studies was assessed using the Cochrane tool in Review Manager 5.4. Each study was evaluated across six domains (selection, performance, detection, attrition, reporting, and other bias), with results summarized in a graphical representation. Each study's risk of bias was ultimately classified as “low risk”, “unclear risk”, or “high risk” (19).

### Statistical analysis

This study employed a network meta-analysis utilizing Stata 17.0 software, specifically leveraging its network and mvmeta packages (20). For continuous variables, the standardized mean difference (SMD) was used as the effect size, with 95% confidence intervals (95% CI) computed. The effect size was derived from the means and standard deviations of baseline and endpoint values for both the intervention and control groups as extracted from the original studies. In instances where the standard deviation of the change value was not reported, it was computed using the formula:


$$\begin{array}{r} \text{SDchange}=\\ \sqrt{\text{S}{\text{D}}^{2}\text{baseline}+\text{S}{\text{D}}^{2}\text{final}-2\times \text{r}\times \text{SDbaseline}\times \text{SDfinal}} \end{array}$$

Where, (r) represents the correlation coefficient between baseline and endpoint values. In the absence of a reported (r), a value of 0.5 was assumed for the analysis, and sensitivity analyses were conducted to evaluate the potential impact of this assumption (21). Stata 17.0 was used to integrate both direct and indirect comparisons of interventions, facilitating ranking and generating network plots, cumulative rank plots, and funnel plots. For networks exhibiting a closed loop, local inconsistency was assessed using the node-splitting method, with a (*P*) value >0.05 indicating acceptable consistency. Inconsistency tests for the network meta-analysis were conducted on closed loops that contained both direct and indirect evidence, quantifying the inconsistency factor (IF) for each loop. An IF value close to 0, with a 95% CI that included 0, suggested a low likelihood of inconsistency. Furthermore, the cumulative rank probability plot area (SUCRA) was used to illustrate the probability that each intervention is the most effective, with values approaching 100% indicating superior efficacy. Publication bias was evaluated through a comparison-adjusted funnel plot, enhancing the robustness of the findings.

### Assessment of the quality of evidence

The certainty of evidence was assessed with the GRADE approach using the GRADEpro GDT online tool (http://gdt.gradepro.org/app#projects). All evidence was rated as high, moderate, low, or very low quality based on standard criteria, ensuring a robust and systematic confidence evaluation for the reported findings.

## Results

**Figure 1:**
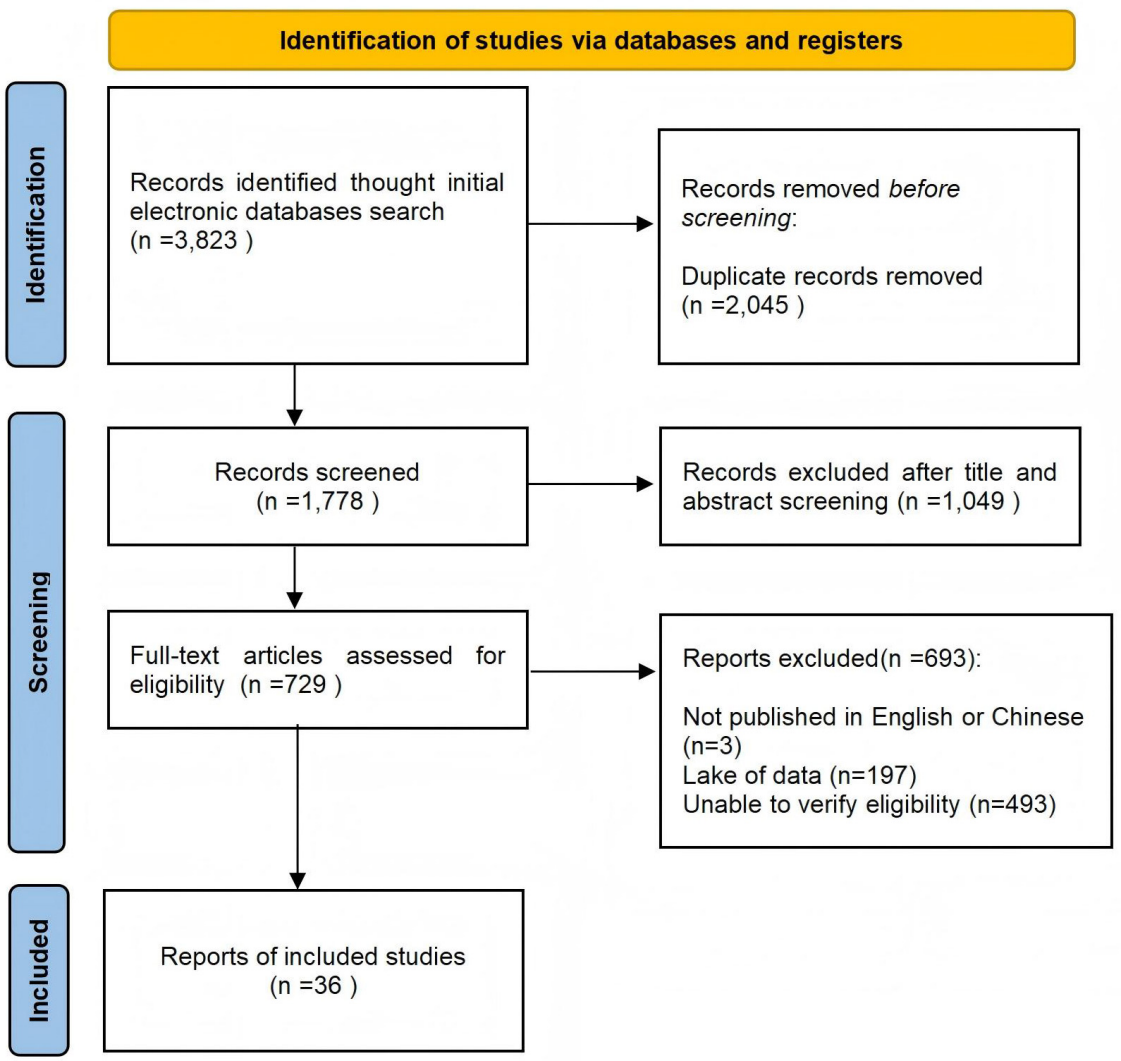
Literature screening flow diagram.

We identified 3,283 articles through initial searches. After the subsequent removal of 2,045 duplicates, 733 articles were excluded upon application of the eligibility criteria. Ultimately, 36 articles were deemed eligible and included in the network meta-analysis. The detailed screening process is depicted in Figure 1.

### Search results

#### Study characteristics

This systematic review synthesized data from 36 RCTs, encompassing a total of 2,731 perimenopausal patients diagnosed with insomnia and concomitant negative affect. The trials collectively evaluated 28 distinct interventions. Insomnia severity, as the primary outcome, was uniformly assessed using the PSQI across all included studies. The measurement of secondary outcomes, specifically negative emotional symptoms, demonstrated considerable heterogeneity in tool selection. The most frequently employed instrument was the KMI, used either alone (*n*=21 studies) or in combination with other scales. Among studies that did not utilize the KMI (*n*=11), various other validated instruments were applied, including the SAS with the SDS, the HAMA with the HAMD, and others such as the GAD-7, PHQ-9, BDI, and BAI. A detailed breakdown of scale utilization by study is provided in Table 2. To ensure methodological consistency, the anatomical localization of all acupuncture points referenced in the included studies conformed to the “Acupoint Naming and Location (GB/T 12346-2006)” standard, as stipulated by the National Standard of the People's Republic of China (2006 edition) (22).

**Table 2:** Randomized controlled trials included in the network meta-analysis.

**Study**	**Age**		**Arm**	**Sample size**		**Interventions**		**Global measures**	**Acupuncture points**
	**Treatment**	**Control**		**Treatment**	**Control**	**Treatment**	**Control**		
Zhang and Fan (56)	52.24 ± 1.86	50.06 ± 1.78	2	23	23	ABA+SP+WM	WM	PSQI, HAMA, HAMD	CV12, CV10, CV6, CV4, GV20, EX-HN1, GV24, EX-HN3
Zhang and Zhang (57)	45.69 ± 4.77	44.78 ± 5.23	2	35	35	RA+WM	WM	PSQI, HAMA	GV26, PC6, LR3, PC7, LI4, LI11, GB34, GB39, ST36, CV6, SP10
Han et al. (58)	51.77 ± 2.37	52.02 ± 2.45	2	38	38	RA+ABA	WM	PSQI, HAMA, HAMD	CV6, CV4, CV12, CV10, CV9, ST25, ST24, SP15, EX-CA1, GB26, EX-HN3, GV24, GB13, EX-HN1, HT7, SP6, KI6, BL62, LR14, LR3
Liu et al. (59)	49.5 ± 3.0	49.6 ± 2.7	2	40	39	AA+WM	WM	PSQI, HAMA, HAMD, Kupperman	Hypothalamus, Endocrine, subcortex, Pituitary gland, Ovary, Internal genitalia, liver, Kidney, Heart, Spleen, Sympathetic nerve, Shenmen, Gonadotropin dots
Wang et al. (60)	49.4 ± 4.99	50.05 ± 3.77	2	25	25	RA	WM	PSQI, HAMA, HAMD	BL23, BL18, ST36, SP6, LR3, CV4
Xue et al. (61)	48.35 ± 2.37	47.75 ± 3.10	2	45	45	RA+WM	WM	PSQI, HAMA, Kupperman	EX-HN1, EX-HN22, GV20, BL62, LI4, ST40, LR14, LR2, LR3, BL18, KI6, SP6, ST36, GV20
Xie et al. (62)	46.44 ± 3.50	46.37 ± 3.50	2	47	46	ABA+WM	WM	PSQI, HAMA	CV12, CV10, CV6, CV4, ST36, ST40, SP6, LR3
Kang (63)	50.45 ± 3.92	49.30 ± 3.15	2	43	43	RA+SP	WM	PSQI, HAMA	BL62, KI6, SP6, BL20, ST36
Zhang et al. (64)	52.76 ± 2.81	52.14 ± 2.63	2	39	39	RA+SP	WM	PSQI, HAMA, HAMD	GV20, HT7, GV24, EX-HN1, GB13, PC6, SP6
Yan et al. (65)	50.8 ± 7.6	49.6 ± 7.2	2	60	60	RA+SP	WM	PSQI, HAMA	EX-HN1, EX-HN22, HT7, SP6, BL18, BL13, GB20, ST36
Zhang et al. (66)	50.45 ± 3.50	48.97 ± 2.88	2	31	31	RA	WM	PSQI, HAMA, HAMD, Kupperman	GV20, EX-HN1, EX-HN22, BL18, BL17, LR3
Zhao et al. (67)	48.94 ± 2.25	48.80 ± 2.25	2	35	35	RA	SH	PSQI, HAMD	EX-HN3, CV20, CV4, CV7, PC6, KI3, LR3, SP6, EX-CA1
Zuo et al. (68)	52.15 ± 2.40	52.20 ± 2.54	2	38	38	RA+WM	WM	PSQI, GDA-7, PHQ-9	GV20, GV24, HT7, EX-HN1, PC6, SP6
Liu et al. (69)	-	-	2	30	30	BAA+XYW	WM	PSQI, SAS, SDS	Insomnia point, Chest Pain point, Abdominal pain point, Headache point
Lin et al. (70)	50 ± 3	50 ± 3	2	36	36	RA	HI	PSQI, SAS, SDS, Kupperman	CV6, CV4, CV12, GV20, GV24, EX-HN3, SP6, ST36, PC6, HT7
Yang et al. (71)	51.4 ± 6.7	52.2 ± 6.2	2	43	45	RA	SH	PSQI, BDI, BAI	CV20, BL23, KI3, EX-HN22
Feng et al. (72)	50.91 ± 4.45	51.03 ± 3.94	2	35	35	CT+EM	EM	PSQI, SAS, SDS	Shenmen, subcortex, Sympathetic nerve, Endocrine, Kidney, Heart, liver, Spleen
Li et al. (73)	52.12 ± 4.19	53.07 ± 3.81	2	42	42	EA	SH	PSQI, SAS, SDS	CV20, GV24, GV29, CV6, CV4, EX-HN22, SP6, HT7, GV4, BL23, KI3, KI7
Hachul et al. (74)	58 ± 4.85	59.8 ± 5.86	2	9	9	RA	SH	PSQI, BDI	-
Zhao et al. (75)	52.1 ± 4.1	51.9 ± 3.9	2	33	33	EA	EA+SP	PSQI, Kupperman	EX-HN 1, GV 24, GB13, CV20, GV 20, BL62, KI6
Hong et al. (76)	45.91 ± 4.41	48.24 ± 4.60	2	45	45	EA	EA+SP	PSQI, Kupperman	EX-HN22, EX-HN1, HT7, SP6, BL62, KI6, KI3, PC7
Lai et al. (77)	52.35 ± 5.39	51.13 ± 5.58	2	40	40	EA+SP	WM	PSQI, Kupperman	EX-HN1, HT7, SP6, LR3, EX-HN22, GB20
Chen et al. (78)	-	-	2	40	40	RA+MT	RA	PSQI, Kupperman	CV4, EX-CA1, ST36, SP6, GV20, EX-HN22, KI1
Lu et al. (79)	48.3 ± 5.0	48.6 ± 4.8	2	30	30	RA+AA	RA	PSQI, Kupperman	BL15, BL23, HT7, KI3, Kidney, Shenmen, Heart, Subcortex, Endocrine, Sympathetic nerve
Guo (80)	49.83 ± 3.65	50.20 ± 4.10	2	30	30	RA	WM	PSQI, Kupperman	EX-HN3, CV6, CV4, CV12, GV20, GV24, SP6, ST36, PC6, HT7
Han et al. (81)	50.0 ± 2.9	50.2 ± 3.8	2	60	60	RA+SP	SP	PSQI, Kupperman	BL15, BL20, BL23, GV20, GV24, GV16, HT7, ST36, SP6, KI6, KI3, LR3
Huang et al. (82)	50.8 ± 4.1	50.2 ± 4.3	2	45	45	ABA+SP	WM	PSQI, Kupperman	CV12, CV10, CV6, CV4, ST24, ST26, KI16, KI13
Liu and Zhang (83)	50.12 ± 3.17	40.56 ± 3.24	2	48	48	RA+SP	SP	PSQI, Kupperman	GV24, GV20, EX-HN22, EX-HN3, EX-HN1, KI3, HT7, GB13, SP6
Chen (84)	50.36 ± 2.21	50.45 ± 2.14	2	33	33	SA+SP	SP	PSQI, Kupperman	GV20, GV22, GV24, GB17
Ge et al. (85)	58.1 ± 4.7	57.6 ± 5.2	2	46	46	EA+AA+WM	WM	PSQI, Kupperman	GV20, GV22, GV24, GB17, Heart, Kidney, Shenmen, Sympathetic nerve, Endocrine system
Gu et al. (86)	49.83 ± 3.84	49.21 ± 2.94	2	45	45	RA+PT	WM	PSQI, Kupperman	GV20, GV2, EX-HN3, EX-HN22, HT7, LI4, ST36, SP6, GB34, TE6, KI3, LR3
Yu et al. (87)	49.7 ± 3.2	48.8 ± 3.4	2	30	30	EA+HI	SH+HI	PSQI, Kupperman	GV20, EX-HN3, ST25, CV4, BL18, BL23, KI3, LR3
Zhang et al. (88)	46.69 ± 5.42	47.12 ± 5.13	2	50	50	RAH	RA	PSQI, Kupperman	BL10, GV20, GV16
Liu et al. (89)	48 ± 2.638	48.14 ± 2.417	2	30	30	RA+SP	WM	PSQI, Kupperman	BL15, BL18, BL23, EX-CA1, SP6, HT7, KI6, EX-HN22, LR2, GB43, GB20, LR3, CV4, GV4, ST40, CV12
Shen et al. (90)	52.85 ± 2.69	52.21 ± 2.32	2	38	38	AA+SP	AA	PSQI, Kupperman	Subcortex, Occiput, Shenmen, Anterior ear lobe, Spleen, kidney, heart, liver
Wei et al. (91)	50.071 ± 2.963	51.20 ± 2.62	2	29	28	TA+BA	RA	PSQI, Kupperman	GV20, EX-HN3, CV12, CV10, CV6, CV4, SP6, KI3, EX-CA1

#### Risk of bias assessment

**Figure 2:**
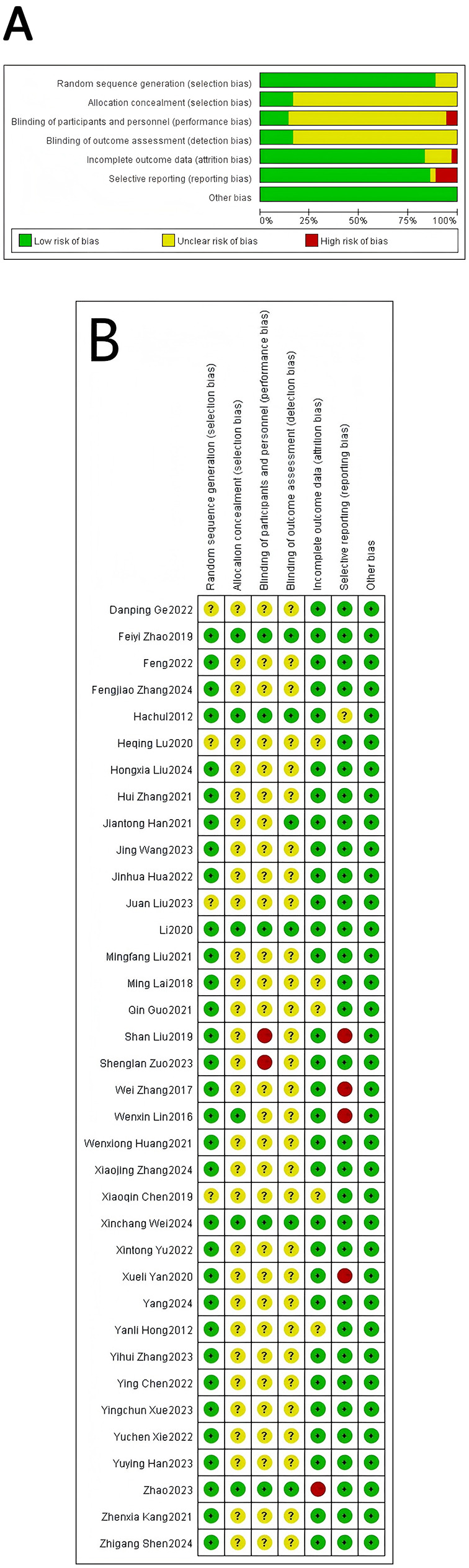
Bias risk assessment. **(A)** Risk of bias overall. **(B)** Risk of bias in individual studies.

The assessment of quality evaluation parameters yielded the following results: (a) Random Sequence Generation: 32 RCTs (88.9%) clearly described their randomization methods, while 4 did not specify the method used. (b) Allocation Concealment: 5 RCTs (13.9%) were rated as low risk, whereas the risk for the remaining 31 studies was classified as unclear. (c) Blinding of Participants and Personnel: Due to the inherent nature of acupuncture intervention, practitioners could not be blinded. Among studies employing sham acupuncture as a control, only 7 RCTs (19.4%) successfully blinded participants. (d) Blinding of Outcome Assessment: 6 RCTs implemented blinding for outcome assessors. (e) Incomplete Outcome Data: 1 RCT was rated as having a high risk of bias due to incomplete outcome data, while the others were classified as low risk. (f) Selective reporting risk was low for 31 RCTs (86.1%) with ChiCTR-registered protocols, unclear for 1 trial, and high for 4 trials due to incompletely reported outcomes. (g) No RCTs reported other sources of bias, resulting in an overall unclear risk rating (see Figure 2).

### Network meta-analysis

#### Network plot

**Figure 3:**
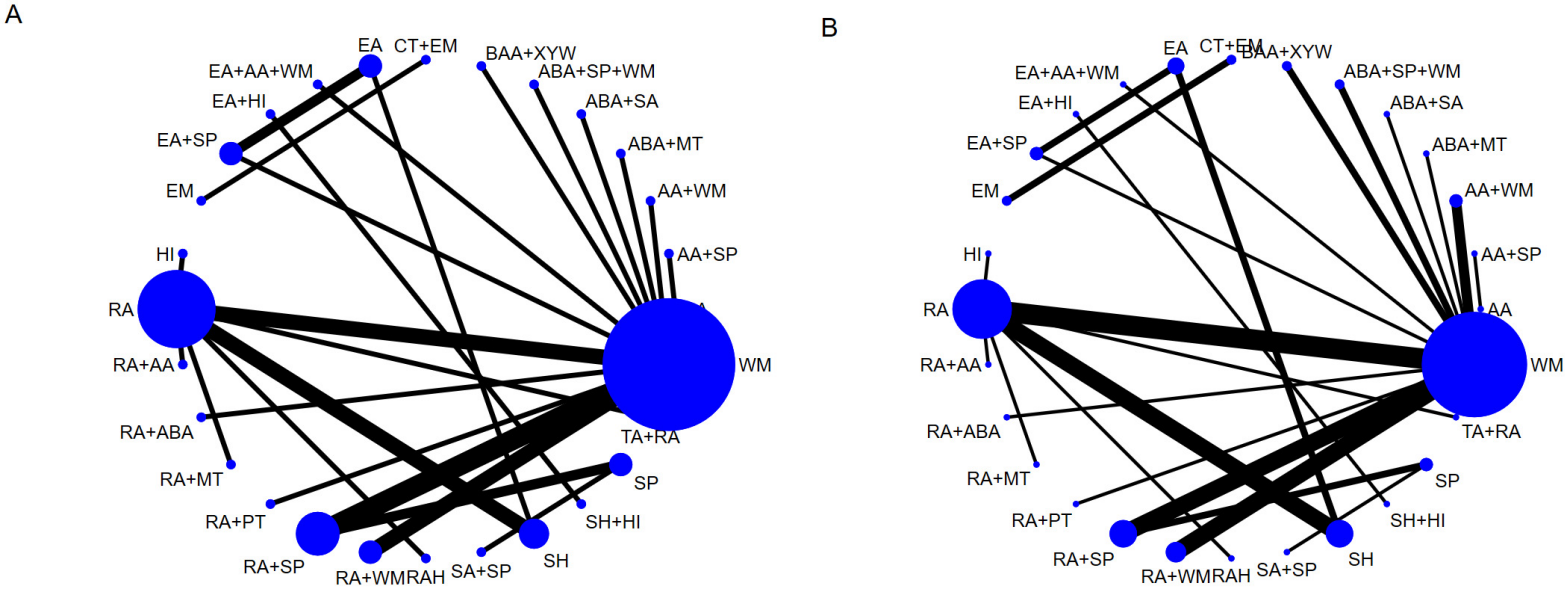
Network plot of interventions under different outcome indicators. **(A)** Network Plot depicting the relationships between interventions based on changes in PSQI scores. **(B)** Network plot depicting the relationships between interventions based on improvements in negative emotion scale scores. The blue nodes represent the interventions included in the analysis, with node size indicating the number of participants using that intervention. The black lines connecting the nodes represent direct comparisons between pairs of interventions, with the thickness of the lines corresponding to the number of studies that have compared the two. A closed loop signifies direct comparisons among three interventions. The black lines connecting the nodes represent direct comparisons between pairs of interventions, with the thickness of the lines corresponding to the number of studies that have compared the two. A closed loop signifies direct comparisons among three interventions.

When using improvements in PSQI scores or negative emotion scale scores as outcome indicators, RA and WM acted as central nodes, with numerous connections to other treatments, exhibiting high centrality. No closed loops were formed (see Figure 3).

#### Consistency test

Following node-splitting analysis, which confirmed the agreement between direct and indirect comparisons (*P* > 0.05), the network meta-analysis was conducted using a consistency model.

#### Results of network meta-analysis

##### psqi-score

**Figure 4:**
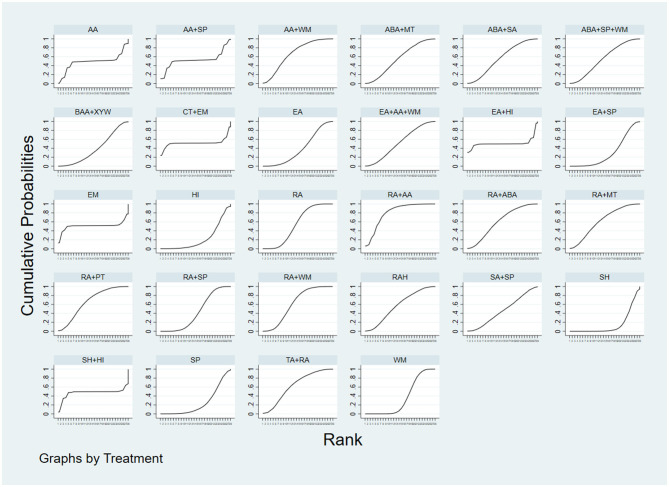
SUCRA plot. The larger area under the curve indicates a higher rank in terms of the intervention's effectiveness.

The synthesis of evidence from the 36 included studies enabled the evaluation of 300 distinct pairwise comparisons among interventions. Notably, RA+AA demonstrated superior efficacy compared to HI, SP, and WM in reducing PSQI scores (*P* < 0.05). RA, RA+MT, RA+PT, RA+WM, RAH, TA+RA, and AA+WM were more effective than SH in decreasing PSQI scores (*P* < 0.05). The remaining comparisons did not show significant differences (*P* > 0.05). Given that lower PSQI scores reflect improved sleep quality, SUCRA rankings were computed, with lower values indicating greater effectiveness. The SUCRA ranking results were as follows: RA+AA > AA+WM > RA+PT > TA+RA > RA+WM > RA+MT > RA+ABA > RAH > ABA+SA > ABA+MT > ABA+SP+WM > RA > AA+SP > CT+EM > EA+AA+WM > EA+HI > EM > AA > SH+HI > SA+SP > RA+SP > EA > BAA+XYW > WM > EA+SP > SP > HI > SH. Importantly, RA+AA exhibited the best performance, demonstrating significant advantages in alleviating insomnia (see Figure 4). Thus, RA+AA constitutes the optimal therapeutic intervention for reducing PSQI scores.

##### negative-mood-scale-scores

**Figure 5:**
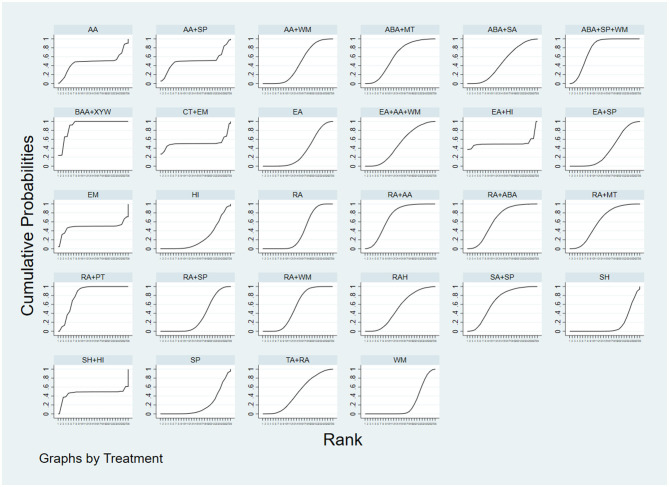
SUCRA plot. The larger area under the curve indicates a higher rank in terms of the intervention's effectiveness.

The network meta-analysis included 36 papers, yielding 378 two-by-two comparisons. Results indicated that, for the improvement of negative emotions, BAA+XYW, ABA+SP+WM, RA+AA, SA+SP, RA+ABA, RA+MT, ABA+MT, RA+WM, and AA were significantly more effective than SH (*P* < 0.05). BAA+XYW, RA+PT, ABA+SP+WM, RA+ABA, SA+SP, RA+AA, and RA+WM were superior to WM (*P* < 0.05). Moreover, BAA+XYW, RA+PT, ABA+SP+WM, RA+ABA, SA+SP, RA+AA, and RA+WM outperformed SP (*P* < 0.05). Among these interventions, RA+AA was notably more effective than the seven interventions, RA, EA, RA+SP, HI, WM, SP, and SH, in reducing negative emotions (*P* < 0.05). Furthermore, among 10 interventions (RA+WM, AA+WM, RA, EA+SP, EA, RA+SP, HI, WM, SP, and SH), ABA+SP+WM significantly outperformed SP in mitigating negative affect (*P* < 0.05). Besides, RA+PT showed greater efficacy in reducing negative affect than 12 interventions (RA+WM, EA+AA+WM, TA+RA, ABA+SA, AA+WM, RA, EA+SP, EA, RA+SP, HI, WM, and SP). The effect of BAA+XYW was particularly pronounced in ameliorating negative emotions (*P* < 0.05). The SUCRA ranking results yielded the following hierarchy of interventions for improving negative affect: BAA+XYW > RA+PT > ABA+SP+WM > RA+AA > SA+SP > RA+ABA > RA+MT > ABA+MT > RA+WM > RAH > EA+HI > CT+EM > AA+SP > EA+AA+WM > SH+HI > TA+RA > EM > AA > ABA+SA > AA+WM > RA > EA+SP > EA > RA+SP > HI > WM > SP > SH. Indeed, BAA+XYW was the most effective intervention for ameliorating negative emotions (see Figure 5). Building upon the above findings, BAA+XYW may represent the most effective intervention for enhancing emotional wellbeing.

#### Publication bias

**Figure 6:**
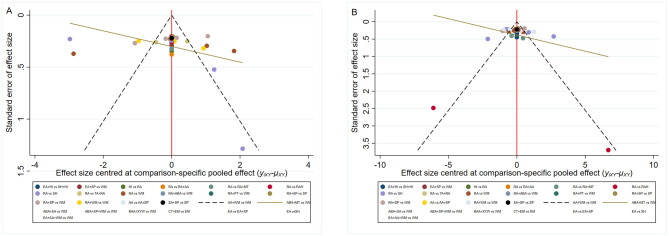
**(A)** Comparison of PSQI scores-corrected funnel plot. **(B)** Comparison of Negative Mood Scale scores-corrected funnel plot.

The funnel plot for PSQI scores as an outcome indicator showed a largely symmetrical distribution, with most points concentrated in the upper-middle region. Some points were located outside the funnel, and one study showed scatter points at the bottom, suggesting potential publication bias and small-sample effects. The distribution of points within the funnel plot for negative emotion scale scores demonstrated a predominant symmetry within the upper inverted triangular region. Nevertheless, the presence of points outside the funnel boundaries at the lower aspect suggests potential influences of publication bias and effects associated with limited sample sizes (see Figure 6). We employed the Trim and Fill method to examine the impact of publication bias on the study results. Using linear estimators for the trimming method analysis, the results indicated that even after accounting for potential publication bias, the combined effect size under the fixed-effects model remained significant (*P* < 0.001). This finding suggests that publication bias has minimal impact on the overall effect size, reinforcing the robustness of the study results.

#### Sensitivity analyses

Sensitivity analyses for both the primary and secondary outcomes were performed using Stata 17.0. The results showed that excluding any single study did not significantly alter the effect sizes of the outcome indicators, suggesting the robustness of the findings.

#### GRADE assessment of evidence quality

**Figure 7:**
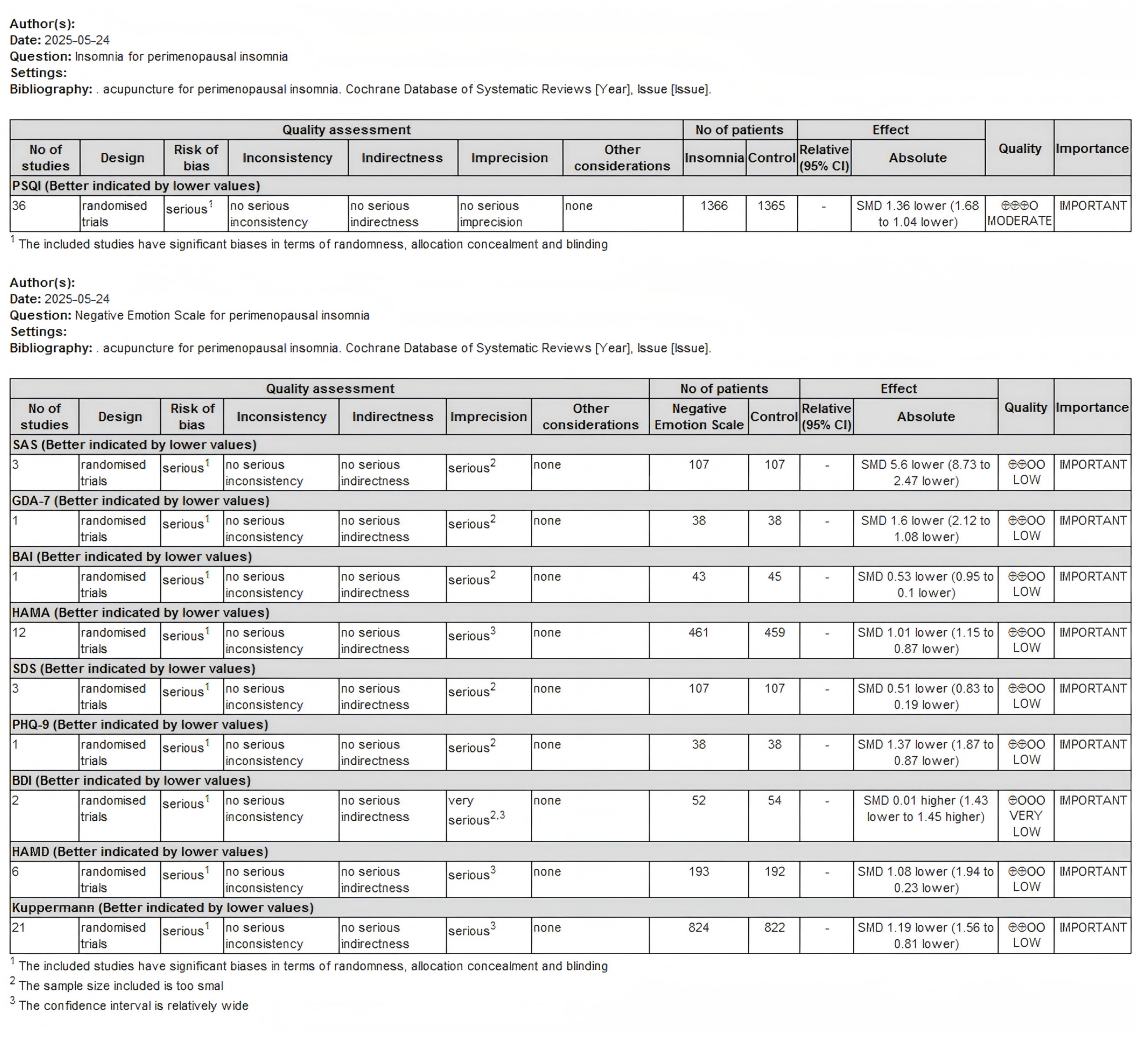
Acupuncture for perimenopausal insomnia with depression GRADE quality of evidence evaluation.

The quality of evidence in this network meta-analysis was evaluated using the GRADE system, which considers five dimensions: study limitations, inconsistency, indirectness, imprecision, and publication bias (23). The certainty of evidence was rated as high, moderate, low, or very low. The GRADE assessment for the outcome measures in this study indicated that the PSQI score was of moderate quality, while the negative emotion scales (SAS, GAD-7, BAI, HAMA, SDS, PHQ-9, BDI, HAMD, Kuppermann) were rated low-quality (see Figure 7).

#### Acupuncture point analysis

**Figure 8:**
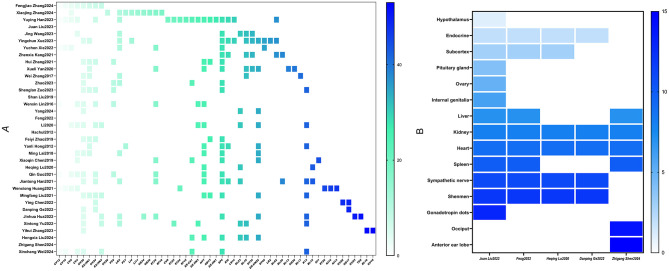
Heatmap of acupoints of the included studies. **(A)** Usage density heatmap of body acupuncture points. **(B)** Usage density heatmap of auricular points.

A heatmap was generated to illustrate the distribution of acupuncture points used in both acupuncture therapy (A) and auricular acupuncture therapy (B) (see Figure 8). The synthesis included 53 body points and 13 auricular points. The analysis of the 36 included studies revealed that, except for the BAA+XYW intervention, the most frequently targeted core points in acupuncture therapy were Sanyinjiao (SP6) and Baihui (GV20). In auricular acupuncture, the heart and kidney points were the most commonly targeted.

## Discussion

### Perimenopause as a transitional window for neuroendocrine disorders, predisposing to insomnia and depression

The perimenopausal period, a critical phase in a woman's life cycle, reportedly affects more than 80% of women with related symptoms (24, 25). Among these, sleep disorders, with a prevalence rate of 50.8%, are the most common and significantly impact quality of life (26). Notably, this period represents a critical window for the onset or worsening of mood disorders and anxiety (27). During this phase, women may frequently experience nighttime awakenings, which can increase the risk of anxiety and depression symptoms, both at night and during the day.

Perimenopause represents a unique and fragile stage in the female life cycle, rendering women particularly susceptible to neuroendocrine disorders. Specifically, due to a sharp decline in estrogen levels, women often experience nocturnal awakenings and poor subjective sleep quality, which in turn increases the risk of anxiety and depressive symptoms (28, 29). Research indicates that this phenomenon primarily stems from the significant drop in estrogen, which weakens the regulation of circadian rhythms by the hypothalamic-pituitary-ovarian axis. Concurrently, hyperactivity of the hypothalamic-pituitary-adrenal axis disrupts the rhythm of cortisol secretion. This dysregulation creates a vicious cycle between nocturnal awakenings and daytime mood disturbances, forming the neurobiological basis for perimenopausal symptoms (30, 31).

Unfortunately, current interventions remain suboptimal. Long-term use of GABAergic hypnotics can lead to increased tolerance and dependency, along with side effects such as headaches and drowsiness (32). Hormone replacement therapy has been associated with heightened risks of venous thrombosis, stroke, gallbladder disease, dementia, and breast cancer (33, 34). Furthermore, current evidence suggests that 25.51% of patients decline medication due to safety concerns (35). This underscores the necessity for non-pharmaceutical alternatives. While acupuncture has demonstrated efficacy, its effectiveness concerning core symptoms of perimenopausal insomnia, such as difficulty falling asleep and maintaining sleep, as well as associated mood disorders, including anxiety and depression, remains unclear and lacks standardization. Therefore, this study employed network meta-analysis to comprehensively evaluate the effectiveness of acupuncture on perimenopausal insomnia and negative emotions.

### Analysis of the multidimensional efficacy of acupuncture interventions: a network meta-analysis in perimenopausal insomnia

#### Insomnia

Network meta-analysis is a sophisticated methodological approach that synthesizes both direct and indirect evidence, facilitating the ranking of various interventions and quantifying the probability of the most effective one (36). In this study, we conducted a comprehensive network meta-analysis of 28 interventions targeting perimenopausal insomnia and associated negative emotions. The findings revealed that the top three interventions for enhancing PSQI scores among perimenopausal women were routine acupuncture combined with auricular acupuncture, auricular acupuncture combined with Western medicine, and routine acupuncture combined with pentatonic therapy. Notably, routine acupuncture combined with auricular acupuncture was identified as the most effective intervention for perimenopausal insomnia.

Acupuncture, as a primary non-pharmacological intervention in traditional Chinese medicine, achieves its therapeutic effects by stimulating specific acupuncture points to restore Qi balance. Its mechanisms are multifaceted, encompassing neuro-regulation, fluid homeostasis, and energy metabolism (37). Hong Zhao et al. found that acupuncture not only increases the concentration of estradiol, but also activates the inhibitory function of the GnRH network to promote the homeostasis of the hypothalamic-pituitary-ovarian axis (38). Meanwhile, a clinical study elucidated the mechanism of acupuncture on the changes in the resting state functional network of the amygdala in patients with premenstrual syndrome (39). The results showed that patients who received acupuncture intervention had significantly enhanced functional connections between the left amygdala and the brainstem, the right hippocampus, and between the left amygdala and the left thalamus, while the functional connection between the left amygdala and the left thalamus showed a weakening trend, thereby enhancing the emotional regulation function of the limbic system (39). Furthermore, numerous studies have shown that acupuncture facilitates the regulation of the nervous system and hormone levels-particularly increasing estradiol and follicle-stimulating hormone-while significantly enhancing cerebral blood circulation and alleviating symptoms such as insomnia, vivid dreams, irritability, and palpitations in perimenopausal women. Notably, the role of serotonin extends beyond regulating rapid eye movement–non-rapid eye movement sleep cycles to encompass a pivotal function in sustaining circadian rhythms and sleep-wake homeostasis (40–42). When examining the efficacy of acupuncture for chronic insomnia and its comorbid anxiety and depressive symptoms, results indicated that patients receiving acupuncture exhibited a reduction in serum cortisol alongside a significant elevation in serotonin levels, with these alterations exceeding those observed in the placebo control group (43). Further analysis revealed that acupuncture demonstrated significant and long-lasting effects in improving sleep quality, sleep efficiency, wake-up time, as well as alleviating anxiety and depression symptoms, and its efficacy was significantly superior to that of placebo treatment (43). These results corroborate the considerable potential of acupuncture on neuroendocrine pathways provides a unique and effective therapeutic strategy for addressing both insomnia and negative emotional symptoms in perimenopause (44–46).

Auricular acupuncture, a specialized form of micro-needle therapy, stimulates specific points on the ear for therapeutic purposes. Its efficacy is grounded in the rich vagus nerve innervation of the auricle and the “inverted fetus” mapping of acupuncture points (47). By targeting auricular points such as Kidney, Shenmen, and Sympathetic, this technique promotes Qi and blood flow, calms the mind, and enhances sleep quality (48). Current evidence suggests that auricular acupuncture may improve sleep architecture by activating cholinergic neurons in the mesencephalon and pontine reticular formation (49). Furthermore, a meta-analysis reported that auricular acupuncture is superior to Western medicine and traditional Chinese medicine in treating insomnia (50). It can significantly reduce the PSQI score, improve clinical efficacy, and is associated with fewer adverse events. Consequently, routine acupuncture combined with auricular acupuncture constitutes a clearly defined and safe non-pharmacological therapeutic intervention for perimenopausal insomnia.

#### Negative emotion

When evaluating improvements in negative emotion scale scores, the top three interventions based on SUCRA rankings were balance acupuncture combined with Xiaoyao powder, routine acupuncture combined with pentatonic therapy, abdominal acupuncture combined with sedative prescriptions and Western medicine. Among these interventions, balance acupuncture in conjunction with Xiaoyao powder demonstrated a statistically significant difference (*P* < 0.05) relative to twenty other comparators, indicating the most extensive therapeutic spectrum. Xiaoyao powder, a traditional Chinese herbal formulation comprising *Bupleurum, Angelica, Atractylodes, Poria, and Licorice*, is widely recognized for its ability to soothe the liver, strengthen the spleen, regulate menstruation, and nourish the blood (51). Research has indicated that Xiaoyao powder increases serum estradiol levels and decreases follicle-stimulating hormone and luteinizing hormone, thereby enhancing ovarian reserve function (52). Furthermore, it upregulates the expression of brain-derived neurotrophic factor and vascular endothelial growth factor in the hippocampus, thereby promoting neuroplastic repair, concurrently mitigating inflammatory responses and modulating neurotransmitter activity (53).

Balance acupuncture operates within an integrative medical model consistent with a “psychological-physiological-social-environmental” framework. It targets the central nervous system by stimulating corresponding peripheral nerves and acupuncture points, aiming to restore systemic balance (54). Selected acupuncture points for insomnia focus on calming the heart, enhancing sleep quality, regulating the autonomic nervous system via the vagus nerve, and alleviating liver Qi stagnation tied to anxiety. Clinical studies substantiate that this modality effectively modulates the hypothalamic-pituitary-gonadal axis and optimizes endocrine profiles, leading to significant improvements in affective symptoms (55). The coordinated regulation of the neuroendocrine network is crucial to balance acupuncture's effectiveness in achieving overall physical and mental equilibrium while alleviating core symptoms.

In summary, the combination of Xiaoyao powder and balance acupuncture has emerged as a promising non-pharmacological intervention for addressing negative emotions during the perimenopausal period. This strategy effectively regulates Qi, blood, organ function, and the neuroendocrine system, addressing psychological factors contributing to sleep disorders and offering a novel perspective for comprehensive insomnia treatment.

### Research limitations and prospects

Despite its contributions, this research was subject to certain limitations that may have affected the strength and application of its evidence. First, the methodological quality of the included studies varied, reflecting differences in study design, sample size, and analytical approaches. Several studies failed to provide a clear description of their randomization procedures and did not report on allocation concealment or blinding, which limits the ability to assess potential biases in their findings. Second, the limited number of studies and participants may have diminished the analytical strength and affected the validity of the findings. Third, a scarcity of longitudinal data in the available randomized trials warrants a guarded interpretation of the outcomes. Fourth, the certainty of the evidence, as evaluated by the GRADE framework, was determined to range from moderate to low. Finally, as most acupuncture approaches are guided by the personalized therapeutic framework of traditional Chinese medicine, natural variations in point selection and needling application may contribute to divergent outcomes across studies.

Given these limitations, future research should prioritize signing more robust, large-scale RCTs with adequate long-term follow-up to further strengthen the evidence base for acupuncture's effectiveness. In clinical practice, when applying the conclusions of this study, decision-makers should consider the individualized nature of acupuncture treatment, patients' specific needs and willingness, and the feasibility and integration potential of different interventions to develop more personalized and comprehensive treatment plans, aiming for optimal therapeutic outcomes.

## Conclusion

This study preliminarily supports the effectiveness of acupuncture through a network meta-analysis and identifies acupuncture treatment options for specific symptoms in perimenopausal women. Our findings suggest that routine acupuncture combined with auricular acupuncture can effectively address sleep disturbances, while a combination of balancing acupuncture and Xiaoyao powder can help alleviate abnormal emotions. These intervention measures provide a precise medical approach distinct from traditional drug therapy for clinical treatment. Nevertheless, given the constraints of the existing evidence base, which is rated as low to moderate by the GRADE assessment, further validation of these findings is necessary through large-sample, multicenter RCTs and studies with extended monitoring. Clinicians are encouraged to integrate these acupuncture treatment options as early as possible for symptomatic perimenopausal women, prioritizing routine acupuncture combined with auricular acupuncture stimulation for insomnia, and using balanced acupuncture combined with Xiaoyao powder for mood disorders.

## Data Availability

The original contributions presented in the study are included in the article/supplementary material, further inquiries can be directed to the corresponding authors.
